# Sex differences in the ventilatory and cardiovascular response to supine and tilted metaboreflex activation

**DOI:** 10.14814/phy2.14041

**Published:** 2019-03-27

**Authors:** Hitesh Joshi, Heather Edgell

**Affiliations:** ^1^ School of Kinesiology and Health Sciences York University Toronto Ontario Canada; ^2^ Muscle Health Research Centre York University Toronto Ontario Canada

**Keywords:** Cerebrovascular, handgrip exercise, post‐exercise circulatory occlusion

## Abstract

Women have attenuated exercise pressor responses compared to men; however, their cerebrovascular and ventilatory responses have not been previously measured. Furthermore, recent evidence has shown that posture change can influence the response of the metaboreflex but this has only been tested in men. Young and healthy men (*n* = 14; age: 21 ± 2) and women (*n* = 11; age: 19 ± 1) underwent 40% MVC static handgrip exercise (HG) for 2 min followed by 3 min of post‐exercise circulatory occlusion (PECO) in the supine and 70° tilted postures. In supine position during HG and PECO only men had an increase in ventilation (Men: Baseline: 12.5 ± 1.7 L/min, HG: 18.6 ± 5.3 L/min, PECO: 17.7 ± 10.3 L/min; Women: Baseline: 12.0 ± 1.5 L/min, HG: 12.4 ± 1.2 L/min, PECO: 11.5 ± 1.3 L/min; Sex × Time interaction *P* = 0.037). In supine position during HG and PECO men and women had similar reductions in cerebrovascular conductance (Men: Baseline: 0.79 ± 0.13 cm/sec/mmHg, HG: 0.68 ± 0.18 cm/sec/mmHg, PECO: 0.61 ± 0.19 cm/s/mmHg; Women: Baseline: 0.87 ± 0.13 cm/sec/mmHg, HG: 0.83 ± 0.14 cm/sec/mmHg, PECO: 0.75 ± 0.17 cm/sec/mmHg; *P* < 0.015 HG/PECO vs. baseline). When comparing the response to PECO in the supine versus upright postures there was a significant attenuation in the increase in mean arterial pressure in both men and women (Supine posture: Men: +23.3 ± 14.5 mmHg, Women: +12.0 ± 7.3 mmHg; Upright posture: Men: +15.7 ± 14.1 mmHg, Women: +7.7 ± 6.7 mmHg; Main effect of sex *P* = 0.042, Main effect of posture *P* < 0.001). Our results indicate sexually dimorphic ventilatory responses to HG and PECO which could be due to different interactions of the metaboreflex and chemoreflex. We have also shown evidence of attenuated metaboreflex function in the upright posture in both men and women.

## Introduction

During exercise, metabolites are produced from active muscle which, in turn, activate afferent nerves resulting in higher sympathetic output, mean arterial pressure, cardiac output, and peripheral blood flow to exercising muscles (Alam and Smirk 1937; McCloskey and Mitchell 1972; Mitchell et al. 1989; Fisher and White 2004; Jarvis et al. 2011; Edgell and Stickland 2014; Incognito et al. 2017; Katayama et al. 2018; Teixeira et al. 2018). Recent studies have established that men have an enhanced sympathetic and pressor response to metaboreflex activation compared to women (Jarvis et al. 2011; Smith et al. 2016; Samora et al. 2019). In groups of men alone and groups where men and women are combined, greater ventilation and middle cerebral artery (MCA) blood flow velocity in response to handgrip and post‐exercise circulatory occlusion (PECO) have been observed (Mitchell et al. 1989; Patrick and Caterisano 2005; Lykidis et al. 2009; Saito et al. 2009; Braz et al. 2014; Edgell and Stickland 2014). During PECO (i.e., metaboreflex activation due to metabolite accumulation), men have reduced MCA conductance with a concurrent decrease in end‐tidal CO_2_ (potentially due to hyperventilation from interactions with the chemoreflex (Edgell and Stickland 2014; Patrick and Caterisano 2005; Saito et al. 2009) or perhaps via a direct influence on control centres in the medulla such as the nucleus tractus solitarius (Sander et al. 2010)) and an increase in blood pressure (Braz et al. 2014; Prodel et al. 2016). However, when hypocapnia was prevented by eucapnic clamping, and mean arterial pressure (and presumably sympathetic activity) remained elevated, there was no reduction in MCA conductance implying that cerebrovascular constriction during PECO was due to changes in CO_2_ only (Braz et al. 2014).

Teixeira et al. (2018) have recently concluded that sitting in an upright posture unloads the cardiopulmonary baroreceptors leading to increased activation of the metaboreflex. Similarly, Ichinose et al. (2017) found that unloading of the carotid baroreflex via a neck pressure device decreases the threshold of activation of the metaboreflex (i.e., greater activation with less stimulus). Since Katayama et al. (2018) found that PECO after 40% MVC handgrip exercise overrides any influence from the cardiopulmonary baroreflex, any influence is likely from the arterial baroreflex. During upright tilt, both the cardiopulmonary and carotid baroreceptors experience unloading due to blood pooling in the lower body and a transient reduction in venous return and mean arterial pressure; however, the sympathetic baroreflex threshold is reset during upright tilt in order to increase total peripheral resistance to protect against hypotension (Schwartz and Stewart 2012). This increase in sympathetic output in upright tilt due to baroreceptor resetting could lead to inhibition of the metaboreflex in the upright posture as Ichinose et al. (2017) suggested that the carotid baroreflex functions to “brake” the metaboreflex and inhibit the pressor response.

Recent studies have found interactions between the baroreflexes and the metaboreflex; however, these studies have included mostly (90%), or exclusively, male participants (Ichinose et al. 2017; Katayama et al. 2018; Teixeira et al. 2018). While there are no sex differences in sympathetic baroreflex function in upright posture (Fu et al. 2009), during exercise (Kim et al. 2012), or during carotid hypotension (Kim et al. 2011), women are known to experience orthostatic hypotension to a greater degree than men (Convertino 1998; Waters et al. 2002; Ganzeboom et al. 2003). The mechanism underlying this susceptibility to orthostatic hypotension in women remains unclear but could potentially involve an attenuation of metaboreflex activity.

This study was performed to investigate (1) sex differences in the ventilatory and cerebrovascular responses to handgrip exercise and post‐exercise circulatory occlusion, and (2) sex differences in the cardiorespiratory response to metaboreflex activation in the upright posture. We hypothesized that since women have an attenuated cardiovascular and sympathetic response to handgrip exercise and post‐exercise occlusion in the supine posture (Jarvis et al. 2011; Samora et al. 2019) then women would also have an attenuated ventilatory response compared to men leading to a smaller reduction in end‐tidal CO_2_. Furthermore, since recent evidence from our lab has found that men and women in the early follicular phase of the menstrual cycle have similar cerebrovascular responses to hypercapnia (Hazlett and Edgell 2018), we hypothesized that the higher end‐tidal CO_2_ would lead to greater cerebrovascular conductance in women. Lastly, we also hypothesized that while both sexes would have a reduced cardiovascular and ventilatory response to metaboreflex activation in the upright posture, the reduction would be greater in women.

## Materials and Methods

### Ethical approval

All protocols were submitted to, and approved by, the Office of Research Ethics at York University (e2017‐275). Participants included in the study provided written approval through informed consent forms. The study conformed to the standards set by the latest revisions of the Declaration of Helsinki.

### Participants

Healthy men (*n* = 14; age: 21 ± 2; BMI: 24 ± 5 kg/m^2^) and women (*n* = 11; age: 19 ± 1; BMI: 22 ± 4 kg/m^2^) with no self‐declared cardiovascular or respiratory conditions were recruited to participate in the study. Women were not taking oral contraceptives, and were tested between days 2–5 of the menstrual cycle (early follicular phase) to minimize the effect of endogenous sex hormones. All subjects were asked to refrain from smoking (none were habitual smokers), caffeine, heavy exercise and eating fatty foods 12 h prior to testing (participants were not fasted).

### Measurements

Continuous blood pressure was taken throughout the test using finger photoplethysmography (NexFin BMEye, Netherlands), and was calibrated to an automated measurement (BPTru Medical Devices, Canada). The hand used for blood pressure measurements was maintained at the level of the heart at all times. Heart rate (HR) was measured with a standard single‐lead electrocardiogram. Stroke volume (SV) was obtained using the Modelflow algorithm from the NexFin, and cardiac output (Q) was determined by multiplying SV and heart rate. Stroke volume index (SVi) and cardiac output index (Qi) were normalized to body surface area. Total peripheral resistance index (TPRi) was calculated as mean arterial pressure/Qi.

Tidal volume (Vt) and respiratory rate (RR) were determined via breathing through a mouthpiece and filter attached to a pneuomotachometer (Hans Rudolph, USA) heated to 37°C. Ventilation (Ve) was calculated as Vt multiplied by RR. End‐tidal carbon dioxide (ETCO_2_) and end‐tidal oxygen (ETO_2_) were measured with CO_2_ and O_2_ gas analyzers and values were calibrated daily according to barometric pressure measurements (Vacumed, USA).

The TOC Neurovision Transcranial Doppler (TCD) ultrasound (Mulitgon Industries Inc., USA) was used to measure brain blood flow velocity through the middle cerebral artery (MCA). A 2‐MHz TCD probe was placed on the right side of the head in the temporal window and held in place by an adjustable headband. Due to technical difficulties with equipment connector lengths, differences in participant height, and the upright position of the tilt table TCD was not adequately acquired in the upright posture. Cerebrovascular Resistance Index (CVRi) was calculated as mean arterial pressure/mean MCA velocity. Cerebrovascular conductance index (CVCi) was calculated as the inverse of CVRi. Resistance index (RI) was calculated as RI = (MCA systolic − MCA diastolic)/MCA systolic. Pulsatility index (PI) was calculated as PI =  (MCA systolic − MCA diastolic)/MCA mean.

### Protocol

At the start of testing, participants were asked to perform two maximum voluntary contractions (MVC; right hand) with a handgrip dynamometer (ADInstruments, USA) and their largest effort was defined as 100%. Participants performed two randomized trials, in a supine or 70° upright tilted posture (Patterson Medical, Canada). Trials were separated by at least 10 min of rest (Men: 20.4 ± 5.6 min; Women: 17.9 ± 2.5 min; *P* = 0.20). The supine trial consisted of 5 min of supine rest, followed by 2 min of static handgrip exercise (approximately 40% of MVC), and then 3 min of post‐exercise circulatory occlusion (PECO). The arm undergoing exercise and occlusion was maintained at the level of the heart at all times. PECO was achieved by inflating a blood pressure cuff placed around the exercising forearm to +40 mmHg above systolic blood pressure approximately 10 sec before the end of exercise. In the tilted trial, 5 min of supine rest was followed by 70° upright tilt. Tilt was maintained for a total of 7 min including 2 min of tilted baseline, 2 min of 40% MVC handgrip exercise, and 3 min of PECO.

### Data analysis

**Figure 1:**
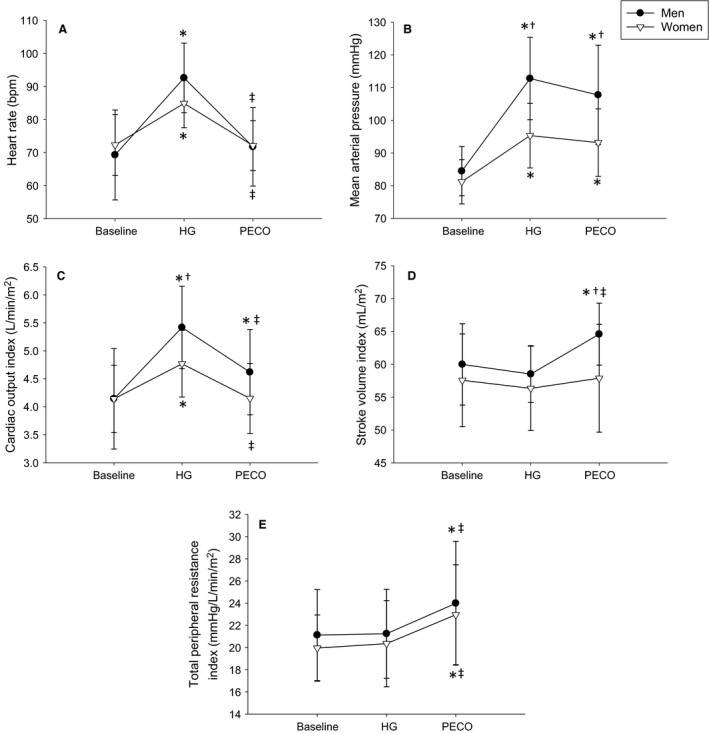
Heart rate (A), mean arterial pressure (B), cardiac output index (C), stroke volume index (D), and total peripheral resistance index (E) responses to handgrip exercise (HG) and post‐exercise circulatory occlusion (PECO) in men (black circles) and women (white triangles). *indicates a significant difference from baseline, ^†^indicates a significant sex difference at a particular timepoint, ^‡^indicates a significant difference from HG.

**Figure 2:**
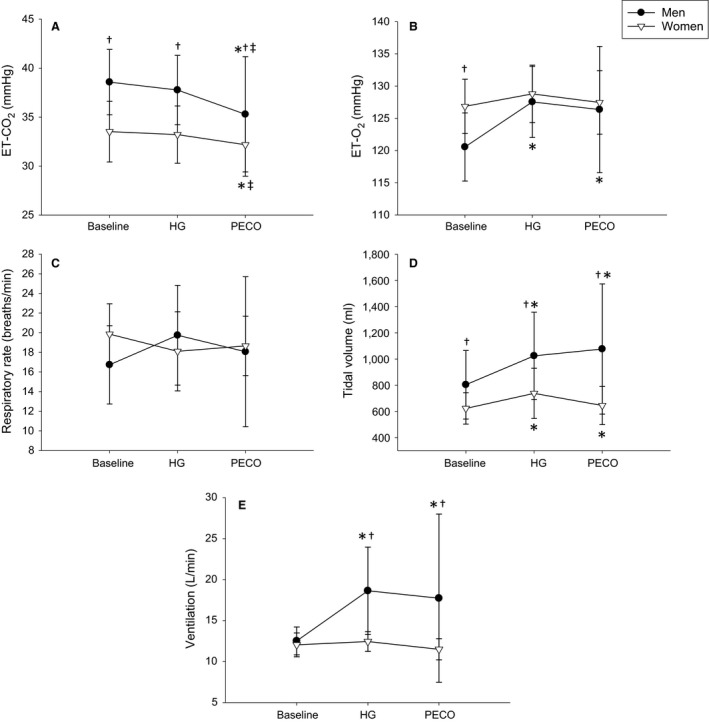
End‐tidal CO_2_ (ETCO_2_; A), end‐tidal O_2_ (ETO_2_; B), respiratory rate (C), tidal volume (D), and ventilation (E) responses to handgrip exercise (HG) and post‐exercise circulatory occlusion (PECO) in men (black circles) and women (white triangles). *indicates a significant difference from baseline, ^†^indicates a significant sex difference at a particular timepoint, ^‡^indicates a significant difference from HG.

**Figure 3:**
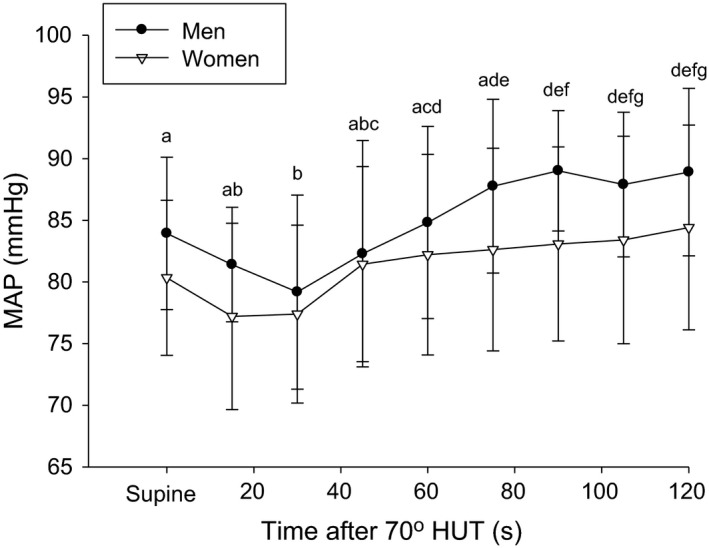
Mean arterial pressure response to the first 2 min of 70° head‐up tilt (HUT) in men (black circles) and women (white triangles). Letters denote similarities between time points.

**Figure 4:**
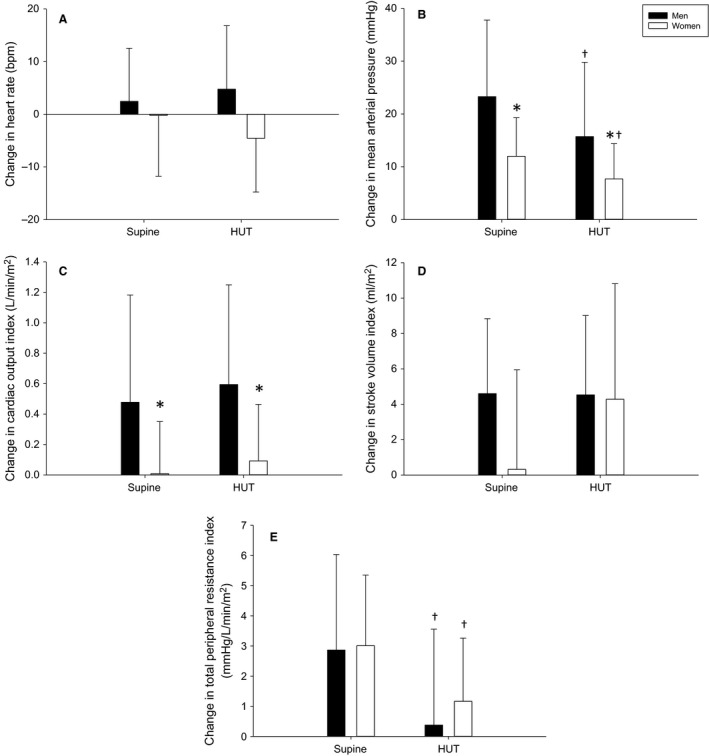
Change in heart rate (A), mean arterial pressure (B), cardiac output index (C), stroke volume index (D), and total peripheral resistance index (E) due to post‐exercise circulatory occlusion (PECO) in the supine and head‐up tilt (HUT) postures in men (black bars) and women (white bars). *indicates a significant sex difference, ^†^indicates a significant difference from supine.

**Figure 5:**
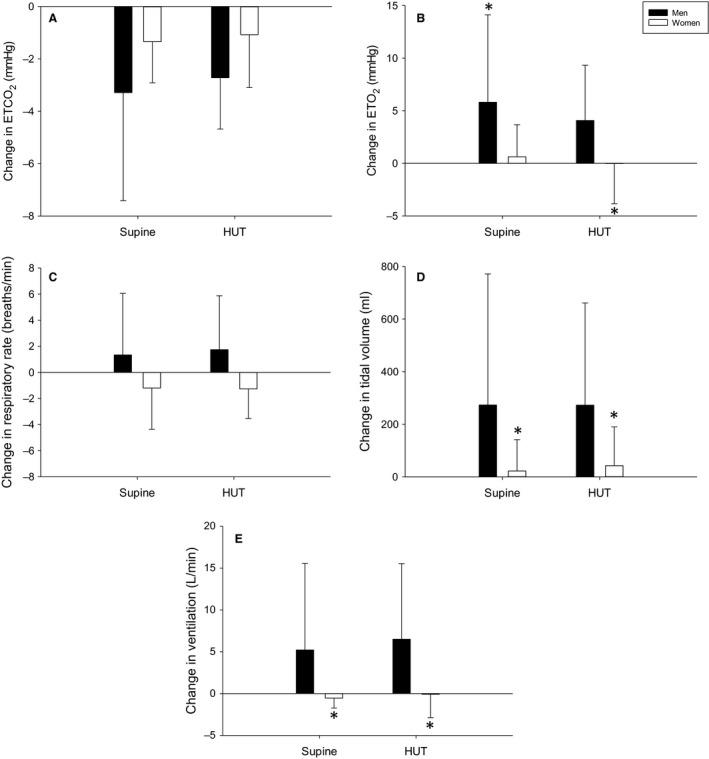
Change in end‐tidal CO_2_ (ETCO_2_; A), end‐tidal O_2_ (ETO_2_; B), respiratory rate (C), tidal volume (D), and ventilation (E) due to post‐exercise circulatory occlusion (PECO) in the supine and head‐up tilt (HUT) postures in men (black bars) and women (white bars). *indicates a significant sex difference.

All signals were obtained using a Powerlab data acquisition device and LabChart software (ADInstruments, USA). Supine one minute averages were obtained at the end of baseline, 2nd minute of HG and 3rd minute of PECO for Figures 1 and 2. Fifteen second averages of mean arterial pressure were taken for the first 2 min of head‐up tilt to ensure achievement of a steady‐state (Fig. 3). Tilted baseline one minute averages were obtained from the 2nd minute of upright tilt (Table 2). The changes due to PECO in the supine and upright postures were calculated for Figures 4 and 5 by subtracting the supine or tilted baseline from the one minute average for supine or tilted PECO, respectively. Supine responses to HG and PECO were compared using a two‐way repeated measures ANOVA (sex and time (repeated) as factors) followed by Tukey post hoc tests (Figures 1 and 2). Sex differences in the tilted baseline averages were determined using unpaired *t*‐tests (Table 2). The response of mean arterial pressure over the first two min of tilt were compared using a two‐way ANOVA (sex and time (repeated) as factors) followed by Tukey post hoc tests (Fig. 3). Comparing the changes due to PECO between postures was completed using a two‐way repeated measure ANOVA (sex and trial (repeated) as factors) followed by Tukey post hoc tests (Figures 4 and 5). Data are presented as mean ± SD and significance was set at *P* < 0.05. Analyses were performed using Sigmaplot 13.0 (Systat Software Inc, USA).

## Results

### Handgrip

Men achieved a maximal voluntary contraction (MVC) of 424 ± 119 N, and women achieved an MVC of 242 ± 72 N. When comparing the 2 MVC attempts that were attempted, there was a difference of 13 ± 10% in men and 14 ± 11% in women. In 80% of our participants the actual MVC was achieved on the first attempt. Over the two min of handgrip exercise (HG) men achieved 35.0 ± 5.5%MVC (137.7 ± 45.8 N) in the supine position and 34.8 ± 3.6%MVC (138.3 ± 38.5 N) in the upright position (%, *P* = 0.80; N, *P* = 0.91) and women achieved 37.1 ± 2.7%MVC (83.6 ± 27.8 N) in the supine position and 35.3 ± 4.2%MVC (87.1 ± 22.3 N) in the upright position (%, *P* = 0.21; *n* = 0.25).

### Supine hemodynamics

All participants increased heart rate (HR) during HG (*P* < 0.001) while HR decreased back to baseline during post‐exercise circulatory occlusion (PECO; *P* > 0.48; Fig. 1A). Men tended to have greater HR during HG compared to women (*P* = 0.076). Both sexes increased mean arterial pressure (MAP) during HG (*P* < 0.001) which remained elevated during PECO (*P* < 0.001). Men had a greater MAP response to HG and PECO compared to women (*P* ≤ 0.002; Fig. 1B). When analyzing the change from baseline, women changed HR by +12.6 ± 5.1 bpm during HG and −0.2 ± 11.6 bpm during PECO, and men changed HR by +23.3 ± 10.1 bpm during HG and +2.5 ± 10.1 bpm during PECO (*P* = 0.001 for sex within HG and PECO). Similarly, when comparing the change from baseline, women changed MAP by +14.1 ± 7.1 mmHg during HG and +12.0 ± 7.3 mmHg during PECO and men changed MAP by +28.3 ± 12.7 mmHg during HG and +23.3 ± 14.5 mmHg during PECO (*P* < 0.01 for sex within HG and PECO).

Men and women had a significant increase in cardiac output index (Qi) during HG (*P* ≤ 0.001) with a greater response in men (*P* = 0.033). During PECO, women decreased Qi back to baseline (*P* = 1.0), yet Qi in men dropped below HG (*P* < 0.001) yet remained above baseline (*P* = 0.006; Fig. 1C). Stroke volume index (SVi) did not change during HG in either sex (*P* > 0.46), and increased during PECO only in men (*P* ≤ 0.002; Fig. 1D). Total peripheral resistance index (TPRi) did not change in either sex during HG (*P* = 0.86), yet increased in both sexes during PECO (*P* < 0.001; Fig. 1E).

### Supine ventilatory measurements

Men had higher end‐tidal CO_2_ (ETCO_2_) at baseline and during HG compared to women (*P* = 0.008), and both sexes decreased ETCO_2_ during PECO compared to baseline and HG (*P* ≤ 0.002; Fig. 2A). Men had lower end‐tidal O_2_ (ETO_2_) compared to women at baseline (*P* = 0.018), but not during HG or PECO (*P* > 0.66) due to a significant increase above baseline (*P* < 0.001; Fig. 2B). There were no significant effects of sex, HG, or PECO on respiratory rate (RR) although there was a trend for an increase in RR during HG in men only (*P* = 0.057; Fig. 2C). Men had higher tidal volume (Vt) compared to women at all time points (*P* = 0.004) and both sexes increased Vt during HG (*P* = 0.041) and PECO (*P* = 0.032) compared to baseline (Fig. 2D). In women, tidal volume changed from 623 ± 120 mL at baseline to 737 ± 192 mL during HG to 645 ± 145 mL during PECO. Importantly, while men and women had similar ventilation (Ve) at baseline (*P* = 0.82), only men increased Ve during HG (*P* = 0.002) and PECO (*P* = 0.003) resulting in higher Ve compared to women at these time points (*P* = 0.004; Fig. 2E).

### Supine cerebrovascular response

Men had higher cerebrovascular resistance index (CVRi) compared to women at all time points (*P* = 0.049; Table 1). In both sexes, HG exercise increased mean and diastolic MCA velocity (*P* ≤ 0.018) while decreasing resistance index (RI), pulsatility index (PI), and cerebrovascular conductance index (CVCi) (*P* ≤ 0.015; Table 1). The response to PECO was also similar in both sexes. PECO reduced mean and diastolic MCA velocity, and increased RI and PI, back to baseline (*P* ≥ 0.23 compared to baseline; Table 1). PECO reduced systolic MCA velocity (*P* = 0.004) and CVCi (*P* < 0.001) below baseline while increasing CVRi (*P* = 0.002) in both sexes (Table 1). The ΔMCAmean/ΔETCO_2_ between supine and PECO was −1.04 ± 7.11 cm/sec/mmHg for men and −4.63 ± 11.9 cm/sec/mmHg for women (*P* = 0.44). The ΔMCAmean/ΔMAP between supine and PECO was −0.06 ± 0.40 cm/sec/mmHg for men and −0.44 ± 0.64 cm/sec/mmHg for women (*P* = 0.13).

**Table 1:** Cerebrovascular response to handgrip exercise and post‐circulatory occlusion in men and women in the supine posture

	Men			Women		
	Baseline	HG	PECO	Baseline	HG	PECO
MCA_mean_ (cm/s)	65.9 ± 11.9	73.2 ± 16.3*	62.3 ± 14.5‡	69.8 ± 9.9	76.7 ± 10.8*	67.2 ± 13.0‡
MCA_systolic_ (cm/s)	110.7 ± 15.6	108.0 ± 24.4	98.1 ± 23.6*	106.6 ± 11.0	106.4 ± 12.3	100.4 ± 13.0*
MCA_diastolic_ (cm/s)	43.2 ± 10.3	50.4 ± 12.8*	40.8 ± 12.2‡	45.8 ± 9.4	52.9 ± 11.3*	45.2 ± 14.2‡
Resistance Index	0.61 ± 0.06	0.53 ± 0.07*	0.58 ± 0.09‡	0.57 ± 0.07	0.50 ± 0.07*	0.56 ± 0.11‡
Pulsatility Index	1.04 ± 0.17	0.79 ± 0.16*	0.94 ± 0.27‡	0.88 ± 0.18	0.71 ± 0.14*	0.86 ± 0.26‡
CVRi (mmHg/cm/sec)†	1.30 ± 0.20	1.56 ± 0.43	1.80 ± 0.68*	1.17 ± 0.15	1.24 ± 0.25	1.39 ± 0.29*
CVCi (cm/sec/mmHg)	0.79 ± 0.13	0.68 ± 0.18*	0.61 ± 0.19*	0.87 ± 0.13	0.83 ± 0.14*	0.75 ± 0.17*

^†^Indicates a sex difference; ^‡^indicates a difference from HG; *indicates a difference from baseline; MCA is middle cerebral artery; CVRi is cerebrovascular resistance index; CVCi is cerebrovascular conductance index; HG is handgrip exercise; PECO is post‐exercise circulatory occlusion. *n* = 10 for men and *n* = 8 for women.

### Upright hemodynamics

After two min of upright tilt women had lower Qi (*P* = 0.005), lower SVi (*P* = 0.005), lower TPRi (*P* = 0.036), lower ETCO_2_ (*P* = 0.025), and lower Vt (*P* = 0.034) compared to men; however, there were no sex differences in HR (*P* = 0.85), MAP (*P* = 0.08), ETO_2_ (*P* = 0.10), Ve (*P* = 0.20) or respiratory rate (*P* = 0.34; Table 2). Men and women had a significant reduction in MAP after 30 sec of head‐up tilt (HUT; *P* = 0.006) which increased back to baseline after 45 (*P* = 1.0), 60 (*P* = 0.92), and 75 sec (*P* = 0.07) of HUT. After 75 sec MAP was significantly higher than baseline (*P* ≤ 0.019), and a new steady‐state MAP was obtained after 60 sec of HUT (75 sec vs. 120 sec, *P* = 0.89). There were no sex differences in the MAP response throughout 120 sec of HUT (*P* = 0.17; Fig. 3).

**Table 2:** Baseline data during upright tilt

	Men	Women
Heart rate (bpm)	86.6 ± 13.0	85.7 ± 9.5
Mean arterial pressure (mmHg)	88.4 ± 5.7	83.1 ± 8.4
Cardiac output index (L/min/m^2^)	4.0 ± 0.8	3.8 ± 0.6*
Stroke volume index (mL/m^2^)	46.0 ± 6.6	44.5 ± 7.9*
Total peripheral resistance index (mmHg/L/min/m^2^)	22.9 ± 3.9	22.4 ± 3.8*
End‐tidal CO_2_ (mmHg)	36.3 ± 4.9	32.2 ± 2.9*
End‐tidal O_2_ (mmHg)	125 ± 6	129 ± 4
Respiratory rate (breath/min)	16.2 ± 4.5	17.7 ± 2.7
Tidal volume (mL)	876 ± 248	687 ± 134*
Ventilation (L/min)	13.2 ± 3.2	11.7 ± 1.9

* Indicates a significant difference between men and women.

There were no differences between the sexes in the HR response to PECO in the supine or upright postures (*P* = 0.10), nor was there an effect of posture (*P* = 0.70; Fig. 4A). Women had a smaller increase in MAP during PECO in both postures compared to men (*P* = 0.041) and the increase in MAP due to PECO was smaller in the head‐up tilt (HUT) position compared to supine (*P* < 0.001; Fig. 4B). Women also had a smaller increase in Qi during PECO both postures compared to men (*P* = 0.032), and there was no influence of HUT on the response to PECO in either sex (*P* = 0.23; Fig. 4C). There was no effect of sex or HUT on the SVi response to PECO (*P* > 0.09; Fig. 4D). Sex did not influence the increase in TPRi due to PECO (*P* = 0.92); however, HUT attenuated this increase in both sexes (*P* = 0.027; Fig. 4E).

### Upright ventilatory measurements

There was no influence of HUT on the ETCO_2_ response to PECO (*P* = 0.50); however, there was a trend for a smaller reduction in ETCO_2_ in women compared to men (*P* = 0.062; Fig. 5A). Women had a smaller increase in ETO_2_ during PECO compared to men (*P* = 0.027) and there was no influence of HUT (*P* = 0.33; Fig. 5B). There was no influence of HUT on the RR response to PECO (*P* = 0.80), yet there was a trend for a larger response in men compared to women (*P* = 0.055; Fig. 5C). Men had a larger Vt response to PECO compared to women (*P* = 0.047), and HUT did not influence the Vt response in either sex (*P* = 0.90; Fig. 5D). Men also had a larger Ve response to PECO compared to women (*P* = 0.031), and HUT did not influence the Ve response in either sex (*P* = 0.53; Fig. 5E).

Notably, the large standard deviations in the male Ve responses were due to a single outlier greater than 2 standard deviations from the mean (Baseline, HG, PECO: Supine: 11.3 to 28.1 to 47.7 L/min; Upright: Baseline: 14.7 to 31.1 to 42.0 L/min). The statistical analysis was also conducted without this outlier and the results were similar.

## Discussion

In support of our hypothesis we have provided evidence that women have an attenuated ventilatory response to HG and PECO, yet despite this, women have similar reductions of ETCO_2_ and similar cerebrovascular conductance compared to men. We have also provided evidence supporting our second hypothesis that the pressor response to PECO is attenuated in the upright posture. However, despite a decreased overall pressor response in women compared to men during PECO the metaboreflex attenuation due to upright posture was equal between the sexes.

Similar to previous results we observed an attenuated blood pressure response to HG and PECO in women compared to men (Jarvis et al. 2011; Samora et al. 2019) implying a reduced metaboreflex function in women. Women are known to have reduced forearm strength compared to men, and indeed in the current study women generated lower handgrip force during exercise compared to men. This could potentially lead to less vascular occlusion during the sustained isometric contraction in women and therefore reduced metabolite accumulation during PECO. However, sex differences in the response to metaboreflex activation have previously been shown to occur independent of muscle mass, force production, or exercise duration (Ettinger et al. 1996). Using the Modelflow algorithm to measure cardiac output and stroke volume (as done in the current investigation), Samora et al. (2019) found increased stroke volume and cardiac output values during PECO in men and women compared to baseline with no sex difference in the response over time. While these results are different than the current investigation, where only men have an increase in stroke volume and cardiac output indices during PECO, this discrepancy could be due to the inaccuracies of using Modelflow to measure cardiac output during static handgrip (Dyson et al. 2010), or alternatively due to posture differences where Samora et al. investigated PECO while seated. Future studies should consider using ultrasound to measure stroke volume and cardiac output. While we found that women have lower cardiac output index and stroke volume index responses to PECO compared to men, both men and women had similar increases of total peripheral resistance index during PECO corresponding to the findings of Samora et al. who found similar total vascular conductance responses. Since Jarvis et al. (2011) found that women had lower sympathetic output with similar vascular transduction during PECO we suggest that other vasoconstrictor hormones, such as vasopressin, could potentially be increasing to a greater degree in women. In men, vasopressin has been shown to increase due to 3 min of handgrip exercise (Nazar et al. 2001). Using 40% MVC HG exercise, Teixeira et al. observed a significant increase in total peripheral resistance during PECO in men (Teixeira et al. 1995) and Kiviniemi et al. observed a significant decrease in systemic vascular conductance in a mixed sex group (Kiviniemi et al. 2017); however, using only 30% MVC HG exercise Incognito et al. did not observe a change in total vascular conductance during PECO in men (Incognito et al. 2012). Therefore, intensity of effort is important to consider in future studies.

Edgell and Stickland (2014) observed that the peripheral chemoreflex was activated secondary to metaboreflex activation in men supporting our findings of elevated ventilation in men during PECO (which has been observed previously in all male groups or groups including mostly men (Braz et al. 2014; Houssiere et al. 2005; Gujic et al. 2007)). However, there are also multiple studies which found that ventilation returns to baseline during PECO following exercise. Lykidis et al. (2009, 2010) found that during 2 min of handgrip (40% MVC) there was no significant increase in ventilation above baseline. Therefore, they also observed no difference in PECO versus baseline. Their results could have been due to the inclusion of equal numbers of men and women, thus attenuating ventilation during exercise and PECO. Bruce and White (2012) found that in men ventilation returned to baseline during PECO following occluded leg exercise (50% MVC isometric plantarflexion, one leg), and similarly Rowell et al. (1976) found that ventilation recovered to pre‐exercise levels after cycling exercise and PECO in both legs. Recent evidence from our lab has shown that men have a limb‐dependent mechanoreflex response to passive limb movement (Fouladi et al. 2018) where men exhibit a greater blood pressure response to arm movement compared to leg movement. Therefore, it is theoretically possible that the decrease in ventilation during PECO in the studies involving leg PECO could be due to limb‐dependent interactions with the chemoreflex. Ventilatory measurements during arm versus leg passive movement are needed to determine this.

The increase in ventilation in men during PECO (supine and upright) could potentially contribute to greater venous return via actions of the respiratory pump and abdominal pressure (Takata et al. 1976), especially in light of previous observations that men use their abdominal muscles more when breathing (Romei et al. 2010). Since abdominal compression has been shown to reduce orthostatic hypotension (Denq et al. 1997) we suggest that the lower ventilation and lower abdominal muscle activation in women could contribute to their higher prevalence of orthostatic intolerance. We hypothesize that the absence of an increase in ventilation in women during PECO could imply that (1) the chemoreflex is not influenced by metaboreflex activation in women, (2) the medullary respiratory centres are not directly activated in women, or (3) due to smaller muscle size (and presumably less metabolite production) women may not have passed a necessary threshold for secondary reflex activation. In support of this latter idea, Cui et al. found that when metabolite concentrations are increased above a certain threshold the secondary activation of the muscle mechanoreceptor is enhanced (Cui et al. 2007) suggesting that metabolite concentrations can indeed influence other autonomic reflexes. In order to make any firm conclusions, studies are needed in women which investigate concurrent chemoreflex and metaboreflex function while measuring cardiorespiratory variables and functional MRI of the medulla. We would also need to compare men and women with similar arm size. The hyperventilation observed in men during HG and PECO is supported by the concurrent reduction in ETCO_2_ and the increase in ETO_2_. Lower ETCO_2_ has previously been observed during PECO in men (Beloka et al. 2008; Braz et al. 2014; Edgell and Stickland 2014; Prodel et al. 2016). ETCO_2_ is also reduced in women during PECO despite the absence of hyperventilation; however, tidal volume does increase slightly but significantly. This slight increase in tidal volume could contribute to a reduction in exhaled CO_2_ according to the Bohr equation, Vd/Vt = PaCO_2_ − PeCO_2_/PaCO_2_, where Vd is dead space volume, Vt is tidal volume, PaCO_2_ is the partial pressure of arterial CO_2_, and PeCO_2_ is the partial pressure of exhaled CO_2_. These ventilatory responses were not influenced by the upright posture in either sex.

While previous studies have used the contralateral MCA for measurements of brain blood flow velocity during handgrip exercise (e.g., (Braz et al. 2014; Rowell et al. 1976)), in the current study we used the ipsilateral MCA to minimize the effect of brain metabolism and somatosensory input (Katayama et al. 2018). Therefore, we are investigating the cerebrovascular response to systemic hemodynamic changes and sympathetic activation. Indeed, Fernandes et al. found that during static handgrip exercise the reduction in cerebral vascular conductance was greater in the ipsilateral internal carotid compared to the contralateral internal carotid, and this was abolished by the α1‐receptor blocker prazosin indicating sympathetic vasoconstriction on the ipsilateral side (Fernandes et al. 1937). Furthermore, Verbree et al. (2016) found a reduction in MCA diameter during rhythmic handgrip exercise using MRI scanning suggesting sympathetic control of large cerebral arteries.

Since Braz et al. (2014) concluded that changes in cerebrovascular conductance during supine HG and PECO were due solely to changes in ETCO_2_ (at least in men), it is unsurprising that we also observed a reduction in conductance with a reduction in ETCO_2_ in both sexes. While previous findings have found greater cerebrovascular reactivity in women compared to men (Kastrup et al. 1997; Deegan et al. 2011; Kastrup et al. 1997) investigated concurrent hypercapnia and hypoxia which are both vasodilatory, and Deegan et al. (2011) performed their studies in the seated position which would have unloaded the cardiopulmonary baroreceptors and increased sympathetic output potentially confounding their results.

In partial support of our second hypothesis, we provided evidence that after achieving a new steady‐state blood pressure in the upright posture (1) the pressor response to metaboreflex activation was attenuated in both men and women, (2) the ventilatory response to metaboreflex activation was unchanged, and (3) the attenuation of the pressor response was equal between the sexes. Using either a neck pressure device (Ichinose et al. 2017) or the seated position (Teixeira et al. 2018) previous studies have suggested that unloading of baroreceptors should increase the function of the metaboreflex. However, in neither of these situations is the baroreflex threshold reset to allow for a higher level of sympathetic output as occurs during upright tilt (Fu et al. 2009; Schwartz and Stewart 2012). Indeed, Hartikainen et al. (1995) found that the relationship between R‐R interval and blood pressure during the pressure overshoot of the Valsalva maneuver did not change between the seated and supine positions implying that the baroreflex threshold is unchanged in the seated position. Therefore, our findings of an attenuated pressor response to metaboreflex activation in the upright position support the concept that activation of the arterial baroreflex acts as a “brake” for the metaboreflex (as proposed by (Ichinose et al. 2017) and (Calbet et al. 2015)). However, we did not observe an attenuation of the ventilatory response to metaboreflex activation. This could be due to the attenuation of the metaboreflex ventilatory response counteracting an augmentation of ventilation due to orthostatic stress (Brunner et al. 1982; Schwartz and Stewart 2012). Lastly, while the lack of sex differences in the metaboreflex attenuation was contrary to our original hypothesis, there is currently little evidence to suggest that interactions between autonomic reflexes should differ between the sexes. This could be due to the dearth of studies including women as a separate group and/or due to the actual absence of sex differences. Furthermore, women in the current study were investigated in the early follicular phase of the menstrual cycle when both estrogen and progesterone are expected to be minimal. Future studies should include women in the late follicular and/or luteal phases to investigate if female sex hormones can affect the interactions between autonomic reflexes.

### Limitations

Our data suggest that participants had increased ventilation at baseline (i.e., ET‐O_2_ was higher than expected, and ET‐CO_2_ was lower than expected). While we did not have a familiarization session with the equipment prior to the day of testing, we did randomize the trials in an attempt to account for the psychological effects of first‐time use of the equipment. We suggest that the greater ventilation experienced by our participants was due to the increased mechanical dead space (~140 mL) due to breathing through a combined pneumotach and respiratory filter at all times.

We did not have a direct measurement of sympathetic activity in this study; however, it has previously been shown that in the supine position both men and women have increased muscle sympathetic nerve activity during HG and PECO compared to baseline (Jarvis et al. 2011). In mixed sex groups, sympathetic activity during HG results in vasoconstriction of the middle cerebral artery (MCA) (Verbree et al. 2016), and greater ETCO_2_ increases the cross‐sectional area of the MCA (Coverdale et al. 2014) indicating that changes in MCA diameter (and that of resistance vessels) in the current study could be influencing brain blood flow. Furthermore, women are known to have reduced neurovascular transduction of sympathetic activity in peripheral vessels (Hart et al. 2011; Briant et al. 2016) suggesting that the MCA of women could theoretically be vasoconstricting less than that of men leading to greater brain blood flow. Therefore, conclusions about sex differences in cerebrovascular conductance should be made with care as the diameter of the MCA could be changing differently between the sexes due to both sympathetic activity and hypocapnia.

Lastly, we did not quantify fitness or training status of our participants. Sprinters or resistance trained participants have been shown to have a greater pressor response to circulatory occlusion after exercise (Patrick and Caterisano 2005; Saito et al. 2009; Amano et al. 2011). Therefore, we recommend that future studies include a cardiopulmonary exercise test and strength measurements for all participants. We also recommend that future studies measure arm size/muscle mass and attempt to control for metabolite retention during exercise and before circulatory occlusion by occluding during the exercise, not afterwards.

## Conclusions

We have provided evidence that only men respond to handgrip exercise and post‐exercise occlusion with hyperventilation potentially indicating secondary chemoreflex activation. However, women still have a small but significant increase in tidal volume resulting in a reduction in end‐tidal CO_2_ and cerebrovascular conductance similar to that of men. Lastly, our results imply that baroreflex resetting in the upright posture suppresses the metaboreflex control of blood pressure equally in men and women.

## Conflict of Interest

The authors declare that they have no conflict of interest.

## References

[phy214041-bib-0001] Alam, M. , and F. H. Smirk . 1937 Observations in man upon a blood pressure raising reflex arising from the voluntary muscles. J. Physiol. 89:372–383. 16994867 10.1113/jphysiol.1937.sp003485 PMC1395054

[phy214041-bib-0002] Amano, T. , M. Ichinose , S. Koga , Y. Inoue , T. Nishiyasu , and N. Kondo . 2011 Sweating responses and the muscle metaboreflex under mildly hyperthermic conditions in sprinters and distance runners. J. Appl. Physiol. 111:524–529. 21659489 10.1152/japplphysiol.00212.2011

[phy214041-bib-0003] Beloka, S. , M. Gujic , G. Deboeck , G. Niset , A. Ciarka , J. F. Argacha , et al. 2008 Beta‐adrenergic blockade and metabo‐chemoreflex contributions to exercise capacity. Med. Sci. Sports Exerc. 40:1932–1938. 18845967 10.1249/MSS.0b013e31817fbe11

[phy214041-bib-0004] Braz, I. D. , C. Scott , L. L. Simpson , E. L. Springham , B. W. Tan , G. M. Balanos , et al. 2014 Influence of muscle metaboreceptor stimulation on middle cerebral artery blood velocity in humans. Exp. Physiol. 99:1478–1487. 25217497 10.1113/expphysiol.2014.081687

[phy214041-bib-0005] Briant, L. J. , A. E. Burchell , L. E. Ratcliffe , N. Charkoudian , A. K. Nightingale , J. F. Paton , et al. 2016 Quantifying sympathetic neuro‐haemodynamic transduction at rest in humans: insights into sex, ageing and blood pressure control. J. Physiol. 594:4753–4768. 27068560 10.1113/JP272167 PMC5009776

[phy214041-bib-0006] Bruce, R. M. , and M. J. White . 2012 Muscle afferent activation causes ventilatory and cardiovascular responses during concurrent hypercapnia in humans. Exp. Physiol. 97:208–218. 22058167 10.1113/expphysiol.2011.061606

[phy214041-bib-0007] Brunner, M. J. , M. S. Sussman , A. S. Greene , C. H. Kallman , and A. A. Shoukas . 1982 Carotid sinus baroreceptor reflex control of respiration. Circ. Res. 51:624–636. 7139881 10.1161/01.res.51.5.624

[phy214041-bib-0008] Calbet, J. A. , J. Gonzalez‐Alonso , J. W. Helge , H. Sondergaard , T. Munch‐Andersen , B. Saltin , et al. 2015 Central and peripheral hemodynamics in exercising humans: leg vs arm exercise. Scand. J. Med. Sci. Sports 25(Suppl 4):144–157. 26589128 10.1111/sms.12604

[phy214041-bib-0009] Convertino, V. A. 1998 Gender differences in autonomic functions associated with blood pressure regulation. Am. J. Physiol. 275(6 Pt 2):R1909–R1920. 9843880 10.1152/ajpregu.1998.275.6.R1909

[phy214041-bib-0010] Convertino, V. A. , D. A. Ludwig , J. J. Elliott , and C. E. Wade . 2001 Evidence for central venous pressure resetting during initial exposure to microgravity. Am. J. Physiol. Regul. Integr. Comp. Physiol. 281:R2021–R2028. 11705789 10.1152/ajpregu.2001.281.6.R2021

[phy214041-bib-0011] Coverdale, N. S. , J. S. Gati , O. Opalevych , A. Perrotta , and J. K. Shoemaker . 2014 Cerebral blood flow velocity underestimates cerebral blood flow during modest hypercapnia and hypocapnia. J. Appl. Physiol. 117:1090–1096. 25012027 10.1152/japplphysiol.00285.2014

[phy214041-bib-1100] Cui, J. , V. Mascarenhas , R. Moradkhan , C. Blaha , and L. I. Sinomway . 2008 Effects of muscle metabolites on resposnes of muscle sympathetic nerve activity to mechanoreceptor(s) stimulation in healthy humans. Am. J. Physiol. Regul. Integr. Comp. Physiol. 294:R458–R466. 18003788 10.1152/ajpregu.00475.2007

[phy214041-bib-0012] Deegan, B. M. , F. A. Sorond , A. Galica , L. A. Lipsitz , G. O'Laighin , and J. M. Serrador . 2011 Elderly women regulate brain blood flow better than men do. Stroke 42:1988–1993. 21566238 10.1161/STROKEAHA.110.605618 PMC7111558

[phy214041-bib-0013] Denq, J. C. , T. L. Opfer‐Gehrking , M. Giuliani , J. Felten , V. A. Convertino , and P. A. Low . 1997 Efficacy of compression of different capacitance beds in the amelioration of orthostatic hypotension. Clin. Auton. Res. 7:321–326. 9430805 10.1007/BF02267725

[phy214041-bib-0014] Dyson, K. S. , J. K. Shoemaker , P. Arbeille , and R. L. Hughson . 2010 Modelflow estimates of cardiac output compared with Doppler ultrasound during acute changes in vascular resistance in women. Exp. Physiol. 95:561–568. 20080867 10.1113/expphysiol.2009.050815

[phy214041-bib-0015] Edgell, H. , and M. K. Stickland . 2014 Activation of the carotid chemoreflex secondary to muscle metaboreflex stimulation in men. Am. J. Physiol. Regul. Integr. Comp. Physiol. 306:R693–R700. 24573180 10.1152/ajpregu.00472.2013

[phy214041-bib-0016] Ettinger, S. M. , D. H. Silber , B. G. Collins , K. S. Gray , G. Sutliff , S. K. Whisler , et al. 1996 Influences of gender on sympathetic nerve responses to static exercise. J. Appl. Physiol. 80:245–251. 8847310 10.1152/jappl.1996.80.1.245

[phy214041-bib-2002] Fernandes, I. A. , J. D. Mattos , M. O. Campos , A. C. Machado , M. P. Rocha , N. G. Rocha , et al. 2016 Selective alpha1‐adrenergic blockage disturbs the regional distribution of cerebral blood flow during static handgrip exercise. Am. J. Physiol. Heart Circ. Physiol. 310:H1541–H1548. 27016578 10.1152/ajpheart.00125.2016

[phy214041-bib-0017] Fisher, J. P. , and M. J. White . 2004 Muscle afferent contributions to the cardiovascular response to isometric exercise. Exp. Physiol. 89:639–646. 15364880 10.1113/expphysiol.2004.028639

[phy214041-bib-0018] Fouladi, B. , H. Joshi , and H. Edgell . 2018 Cardiovascular and autonomic responses to passive arm or leg movement in men and women. Eur. J. Appl. Physiol. 119:551–559. 30446863 10.1007/s00421-018-4030-9

[phy214041-bib-0019] Fu, Q. , K. Okazaki , S. Shibata , R. P. Shook , T. B. VanGunday , M. M. Galbreath , et al. 2009 Menstrual cycle effects on sympathetic neural responses to upright tilt. J. Physiol. 587(Pt 9):2019–2031. 19237424 10.1113/jphysiol.2008.168468 PMC2689340

[phy214041-bib-0020] Fu, Q. , B. Verheyden , W. Wieling , and B. D. Levine . 2012 Cardiac output and sympathetic vasoconstrictor responses during upright tilt to presyncope in healthy humans. J. Physiol. 590:1839–1848. 22331415 10.1113/jphysiol.2011.224998 PMC3573307

[phy214041-bib-0021] Ganzeboom, K. S. , N. Colman , J. B. Reitsma , W. K. Shen , and W. Wieling . 2003 Prevalence and triggers of syncope in medical students. Am. J. Cardiol. 91:1006–1008, A8. 12686351 10.1016/s0002-9149(03)00127-9

[phy214041-bib-0022] Gujic, M. , D. Laude , A. Houssiere , S. Beloka , J. F. Argacha , D. Adamopoulos , et al. 2007 Differential effects of metaboreceptor and chemoreceptor activation on sympathetic and cardiac baroreflex control following exercise in hypoxia in human. J. Physiol. 585(Pt 1):165–174. 17884922 10.1113/jphysiol.2007.141002 PMC2375466

[phy214041-bib-0023] Hart, E. C. , N. Charkoudian , B. G. Wallin , T. B. Curry , J. Eisenach , and M. J. Joyner . 2011 Sex and ageing differences in resting arterial pressure regulation: the role of the beta‐adrenergic receptors. J. Physiol. 589(Pt 21):5285–5297. 21859824 10.1113/jphysiol.2011.212753 PMC3225680

[phy214041-bib-0024] Hartikainen, J. , E. Vanninen , and E. Lansimies . 1995 Effect of posture on baroreflex sensitivity in healthy subjects. Clin. Physiol. 15:571–579. 8590552 10.1111/j.1475-097x.1995.tb00545.x

[phy214041-bib-0025] Hazlett, C. , and H. Edgell . 2018 Chemoreflex function and brain blood flow during upright posture in men and women. Physiol Rep. 6:e13571. 10.14814/phy2.13571 PMC5789659 29333725

[phy214041-bib-0026] Houssiere, A. , B. Najem , A. Ciarka , S. Velez‐Roa , R. Naeije , and P. van de Borne . 2005 Chemoreflex and metaboreflex control during static hypoxic exercise. Am. J. Physiol. Heart Circ. Physiol. 288:H1724–H1729. 15604123 10.1152/ajpheart.01043.2004

[phy214041-bib-0027] Ichinose, M. , T. Ichinose‐Kuwahara , K. Watanabe , N. Kondo , and T. Nishiyasu . 2017 The carotid baroreflex modifies the pressor threshold of the muscle metaboreflex in humans. Am. J. Physiol. Heart Circ. Physiol. 313:H650–H657. 28687588 10.1152/ajpheart.00816.2016

[phy214041-bib-0028] Incognito, A. V. , C. J. Doherty , J. B. Lee , M. J. Burns , and P. J. Millar . 2017 Ischemic preconditioning does not alter muscle sympathetic responses to static handgrip and metaboreflex activation in young healthy men. Physiol Rep. 5:e13342. 28720715 10.14814/phy2.13342 PMC5532483

[phy214041-bib-0029] Jarvis, S. S. , T. B. VanGundy , M. M. Galbreath , S. Shibata , K. Okazaki , M. F. Reelick , et al. 2011 Sex differences in the modulation of vasomotor sympathetic outflow during static handgrip exercise in healthy young humans. Am. J. Physiol. Regul. Integr. Comp. Physiol. 301:R193–R200. 21508291 10.1152/ajpregu.00562.2010 PMC3129874

[phy214041-bib-0030] Kastrup, A. , C. Thomas , C. Hartmann , and M. Schabet . 1997 Sex dependency of cerebrovascular CO2 reactivity in normal subjects. Stroke 28:2353–2356. 9412613 10.1161/01.str.28.12.2353

[phy214041-bib-0031] Katayama, K. , J. Kaur , B. E. Young , T. C. Barbosa , S. Ogoh , and P. J. Fadel . 2018 High intensity muscle metaboreflex activation attenuates cardiopulmonary baroreflex‐mediated inhibition of muscle sympathetic nerve activity. J. Appl. Physiol. 125:812–819. 29672226 10.1152/japplphysiol.00161.2018 PMC6842875

[phy214041-bib-0032] Kim, A. , S. H. Deo , J. P. Fisher , and P. J. Fadel . 2012 Effect of sex and ovarian hormones on carotid baroreflex resetting and function during dynamic exercise in humans. J. Appl. Physiol. 112:1361–1371. 22267388 10.1152/japplphysiol.01308.2011 PMC3331588

[phy214041-bib-7007] Kim, A. , S. H. Deo , L. C. Vianna , G. M. Balanos , D. Hartwich , J. P. Fisher , et al. 2011 Sex differences in carotid baroreflex control of arterial blood pressure in humans: relative contribution of cardiac output and total vascular conductance. Am. J. Physiol. Heart Circ. Physiol. 301:H2454–H2465. 21963834 10.1152/ajpheart.00772.2011 PMC3233807

[phy214041-bib-5005] Kiviniemi, A. M. , M. F. Frances , M. Rachinsky , R. Craen , R. J. Petrella , H. V. Huikuri , et al. 2012 Non‐alpha‐adrenergic effects on systemic vascular conductance during lower‐body negative pressure, static exercise and muscle metaboreflex activation. Acta Physiol. (Oxf) 206:51–61. 22591110 10.1111/j.1748-1716.2012.02447.x

[phy214041-bib-0033] Lykidis, C. K. , P. Kumar , and G. M. Balanos . 2009 The respiratory responses to the combined activation of the muscle metaboreflex and the ventilatory chemoreflex. Adv. Exp. Med. Biol. 648:281–287. 19536491 10.1007/978-90-481-2259-2_32

[phy214041-bib-0034] Lykidis, C. K. , P. Kumar , L. C. Vianna , M. J. White , and G. M. Balanos . 2010 A respiratory response to the activation of the muscle metaboreflex during concurrent hypercapnia in man. Exp. Physiol. 95:194–201. 19801386 10.1113/expphysiol.2009.049999

[phy214041-bib-0036] McCloskey, D. I. , and J. H. Mitchell . 1972 Reflex cardiovascular and respiratory responses originating in exercising muscle. J. Physiol. 224:173–186. 5039977 10.1113/jphysiol.1972.sp009887 PMC1331532

[phy214041-bib-0037] Mitchell, J. H. , D. R. Jr Reeves , H. B. Rogers , and N. H. Secher . 1989 Epidural anaesthesia and cardiovascular responses to static exercise in man. J. Physiol. 417:13–24. 2621589 10.1113/jphysiol.1989.sp017787 PMC1189252

[phy214041-bib-3003] Nazar, K. , D. Jezova , and E. Kowalik‐Borowka . 1989 Plasma vasopressin, growth hormone and ACTH responses to static handgrip in healthy subjects. Eur. J. Appl. Physiol. Occup. Physiol. 58:400–404. 2537720 10.1007/BF00643516

[phy214041-bib-0038] Patrick, B. T. , and A. Caterisano . 2005 Hemodynamic adjustments to circulatory arrest during and following isometric handgrip in resistance trained and untrained men. J. Sports Med. Phys. Fitness 45:393–400. 16230992

[phy214041-bib-0039] Prodel, E. , G. M. Balanos , I. D. Braz , A. C. Nobrega , L. C. Vianna , and J. P. Fisher . 2016 Muscle metaboreflex and cerebral blood flow regulation in humans: implications for exercise with blood flow restriction. Am. J. Physiol. Heart Circ. Physiol. 310:H1201–H1209. 26873971 10.1152/ajpheart.00894.2015

[phy214041-bib-4004] Romei, M. , A. L. Mauro , M. G. D'Angelo , A. C. Turconi , N. Bresolin , A. Pedotti , et al. 2010 Effects of gender and posture on thoraco‐abdominal kinematics during quiet breathing in healthy adults. Resp. Physiol. Neurobiol. 172:184–191. 10.1016/j.resp.2010.05.018 20510388

[phy214041-bib-0040] Rowell, L. B. , L. Hermansen , and J. R. Blackmon . 1976 Human cardiovascular and respiratory responses to graded muscle ischemia. J. Appl. Physiol. 41:693–701. 993157 10.1152/jappl.1976.41.5.693

[phy214041-bib-0041] Ruzieh, M. , A. Baugh , O. Dasa , R. L. Parker , J. T. Perrault , A. Renno , et al. 2017 Effects of intermittent intravenous saline infusions in patients with medication‐refractory postural tachycardia syndrome. J. Interv. Card. Electrophysiol. 48:255–260. 28185102 10.1007/s10840-017-0225-y

[phy214041-bib-0042] Saito, M. , S. Iwase , and T. Hachiya . 2009 Resistance exercise training enhances sympathetic nerve activity during fatigue‐inducing isometric handgrip trials. Eur. J. Appl. Physiol. 105:225–234. 18941773 10.1007/s00421-008-0893-5

[phy214041-bib-0043] Samora, M. , A. L. Teixeira , J. L. Sabino‐Carvalho , and L. C. Vianna . 2019 Spontaneous cardiac baroreflex sensitivity is enhanced during post‐exercise ischemia in men but not in women. Eur. J. Appl. Physiol. 119:103–111. 30293100 10.1007/s00421-018-4004-y

[phy214041-bib-0044] Sander, M. , V. G. Macefield , and L. A. Henderson . 2010 Cortical and brain stem changes in neural activity during static handgrip and postexercise ischemia in humans. J. Appl. Physiol. 108:1691–1700. 20185626 10.1152/japplphysiol.91539.2008

[phy214041-bib-0045] Schwartz, C. E. , and J. M. Stewart . 2012 The arterial baroreflex resets with orthostasis. Front. Physiol. 3:461. 23233840 10.3389/fphys.2012.00461 PMC3516802

[phy214041-bib-0046] Smith, J. R. , R. M. Broxterman , S. M. Hammer , A. M. Alexander , K. D. Didier , S. P. Kurti , et al. 2016 Sex differences in the cardiovascular consequences of the inspiratory muscle metaboreflex. Am. J. Physiol. Regul. Integr. Comp. Physiol. 311:R574–R581. 27488888 10.1152/ajpregu.00187.2016

[phy214041-bib-6006] Takata, M. , R. A. Wise , and J. L. Robotham . 1990 Effects of abdominal pressure on venous return: abdominal vascular zone conditions. J. Appl. Physiol. 69:1961–1972. 2076989 10.1152/jappl.1990.69.6.1961

[phy214041-bib-0047] Teixeira, A. L. , M. Daher , M. C. Souza , P. S. Ramos , J. P. Fisher , and L. C. Vianna . 2018 Sympathetically mediated cardiac responses to isolated muscle metaboreflex activation following exercise are modulated by body position in humans. Am. J. Physiol. Heart Circ. Physiol. 314:H593–H602. 29351473 10.1152/ajpheart.00576.2017

[phy214041-bib-0048] Thieben, M. J. , P. Sandroni , D. M. Sletten , L. M. Benrud‐Larson , R. D. Fealey , S. Vernino , et al. 2007 Postural orthostatic tachycardia syndrome: the Mayo clinic experience. Mayo Clin. Proc. 82:308–313. 17352367 10.4065/82.3.308

[phy214041-bib-0049] Verbree, J. , A. Bronzwaer , M. A. van Buchem , M. Daemen , J. J. van Lieshout , and M. van Osch . 2016 Middle cerebral artery diameter changes during rhythmic handgrip exercise in humans. J. Cereb. Blood Flow Metab.. 37:2921–2927. 27837189 10.1177/0271678X16679419 PMC5536799

[phy214041-bib-0050] Waters, W. W. , M. G. Ziegler , and J. V. Meck . 2002 Postspaceflight orthostatic hypotension occurs mostly in women and is predicted by low vascular resistance. J. Appl. Physiol. 92:586–594. 11796668 10.1152/japplphysiol.00544.2001

